# Logical modelling of *in vitro* differentiation of human monocytes into dendritic cells unravels novel transcriptional regulatory interactions

**DOI:** 10.1098/rsfs.2020.0061

**Published:** 2021-06-11

**Authors:** Karen J. Nuñez-Reza, Aurélien Naldi, Arantza Sánchez-Jiménez, Ana V. Leon-Apodaca, M. Angélica Santana, Morgane Thomas-Chollier, Denis Thieffry, Alejandra Medina-Rivera

**Affiliations:** ^1^ Laboratorio Internacional de Investigación sobre el Genoma Humano, Universidad Nacional Autónoma de México, Juriquilla, México; ^2^ Computational Systems Biology team, Institut de Biologie de l’École Normale Supérieure, Inserm, CNRS, Université PSL, Paris, France; ^3^ Centro de Investigación en Dinámica Celular (IICBA), Universidad Autónoma del Estado de Morelos, Cuernavaca, México

**Keywords:** dendritic cells, differentiation, logical modelling, regulatory networks

## Abstract

Dendritic cells (DCs) are the major specialized antigen-presenting cells, thereby connecting innate and adaptive immunity. Because of their role in establishing adaptive immunity, they constitute promising targets for immunotherapy. Monocytes can differentiate into DCs *in vitro* in the presence of colony-stimulating factor 2 (CSF2) and interleukin 4 (IL4), activating four signalling pathways (MAPK, JAK/STAT, NFKB and PI3K). However, the downstream transcriptional programme responsible for DC differentiation from monocytes (moDCs) remains unknown. By analysing the scientific literature on moDC differentiation, we established a preliminary logical model that helped us identify missing information regarding the activation of genes responsible for this differentiation, including missing targets for key transcription factors (TFs). Using ChIP-seq and RNA-seq data from the Blueprint consortium, we defined active and inactive promoters, together with differentially expressed genes in monocytes, moDCs and macrophages, which correspond to an alternative cell fate. We then used this functional genomic information to predict novel targets for previously identified TFs. By integrating this information, we refined our model and recapitulated the main established facts regarding moDC differentiation. Prospectively, the resulting model should be useful to develop novel immunotherapies targeting moDCs.

## 1. Introduction

Dendritic cells (DCs) are the main antigen-presenting cells [1]. By presenting antigens to the naïve lymphocytes, they initiate the immune response against various kinds of pathogens [2]. This capacity of DCs to activate the adaptive immune response opens interesting prospects for immunotherapies [3]. Circulating in the peripheral blood, monocytes are easily accessible and can be differentiated into dendritic cells, called moDCs (for monocyte-derived DCs), using an established protocol [4].

The protocol to differentiate monocytes into moDCs consists in cultivating monocytes with colony-stimulating factor 2 (CSF2) and interleukin 4 (IL4) [4,5]. When only IL4 is used, monocytes are activated, while their treatment with only CSF2 results in their differentiation into macrophages. Only the combination of both stimuli enables DC differentiation, pointing to the importance of signalling interplay for the differentiation of moDCs. It is known that CSF2 signalling leads to the activation of NF*Κ*B, MAPK, PI3K, JAK2 and STAT5 [6,7]. By contrast, IL4 signalling activates the JAK/STAT pathway, with JAK1 activating STAT3, and JAK3 activating STAT6 [8]. There are several well-known transcription factors (TFs) that are ultimately activated by CSF2 and IL4 signalling pathways, but only a fraction of the target genes participating in moDC differentiation have been reported [7,9–11].

To integrate multiple signalling pathways into a comprehensive regulatory network and check its coherence with existing expression data, one can rely on the construction of a dynamical model [12]. As most of the available data are qualitative, we opted for using a qualitative approach. Logical modelling is well suited to represent such qualitative data and has been applied to similar processes [13–15]. This qualitative formalism relies on the construction of a regulatory graph, whose nodes denote molecular components (e.g. genes, proteins and lncRNA), while signed arcs denote positive or negative (or sometimes dual) regulatory interactions. In the simplest cases, nodes are associated with Boolean variables, which take the values 0 or 1, denoting the absence/inactivity or the presence/activation of the corresponding component, respectively [16]. When needed, multilevel variables (e.g. ternary variables taking the values 0, 1, 2) can be used to account for different ranges of activation (e.g. negligible, medium and high). Logical models are usually derived from a careful manual curation of relevant scientific literature, but they can also be enriched with other sources of information, such as high-throughput sequencing data [17].

GINsim is a computational software dedicated to the building and analysis of logical models of cellular networks [16]. GINsim includes specific tools to perform model simulations, as well as efficient algorithms to identify the attractors of the system (stable states and/or oscillatory behaviour), for wild-type or mutant conditions [16]. The resulting model can be further analysed using the CoLoMoTo suite, an interactive toolbox integrating several logical modelling software tools, with a uniform interface to perform complementary analyses, which are easy to share and reproduce through the use of notebooks [18].

The aim of our study was to integrate all the information gathered from scientific literature and functional genomic data (RNA-seq and ChIP-seq) into a logical model of the regulatory network underlying moDC differentiation. We further included key macrophage differentiation elements to complement our study [11,19,20]. The resulting model recapitulates the salient cell commitment features for each of the initial conditions considered: (i) IL4 alone fosters monocyte activation, (ii) CSF2 alone fosters macrophage commitments, while (iii) CSF2 and IL4 together foster moDC commitment, with the corresponding typical gene expression patterns.

## 2. Results

### 2.1. Information gathered from literature curation leads to a fragmentary model for the differentiation of monocytes into dendritic cells

**Figure 1.**
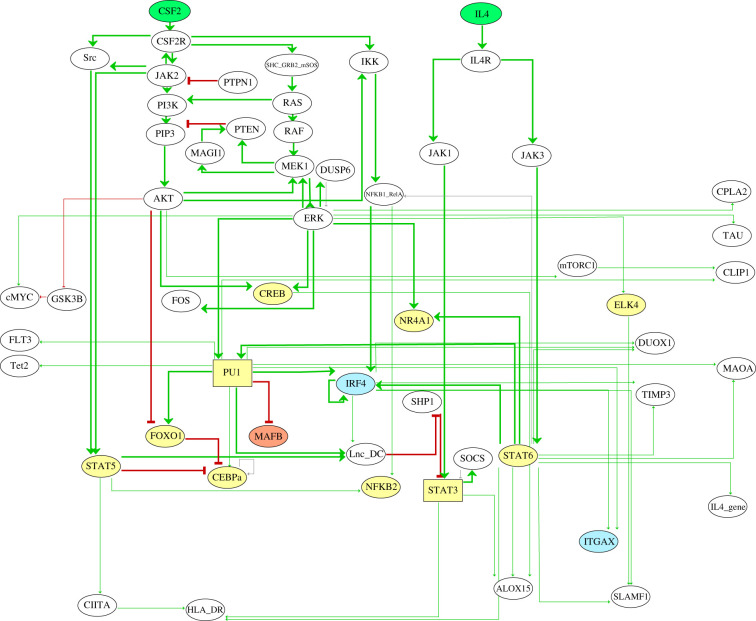
Regulatory graph controlling monocyte to moDC differentiation, as derived from the scientific literature. Ellipsoid nodes are associated with Boolean components (0 and 1), whereas the two rectangular nodes are associated with ternary components (0, 1 and 2). The green nodes at the top represent the inputs (CSF2 and IL4), the yellow nodes denote transcription factors, the blue nodes denote moDC-specific genes, while orange nodes denote macrophage-specific genes. Nodes left in white correspond to components of generic signalling pathways. Green and red arcs denote positive and negative interactions, respectively. The core network interactions are emphasized by thicker arcs.

To better understand the regulatory network controlling moDCs differentiation, we analysed the scientific literature and integrated relevant information into a regulatory graph. In this process, we focused on monocyte to moDC differentiation studies carried primarily on human cells, in particular on experiments where CSF2 and IL4 were used alone or in combination in otherwise similar culture conditions. The resulting regulatory graph is shown in figure 1.

Based on this first regulatory graph, we used GINsim to define logical rules, combining conditions on regulatory nodes with NOT, AND and OR Boolean operators, to compute the corresponding stable states, and to perform simulations in order to determine the cellular phenotypes reached for each specific input condition. Note that although most nodes are associated with Boolean variables, we assigned ternary variables to the model components STAT3 and PU1. In such a situation, two non-overlapping rules are defined, enabling the value 1 and 2, respectively (see electronic supplementary material, table S1).

For this preliminary model, we obtained six stable states, but only one of them could be directly interpreted as a cellular phenotype (pre-DCs), while the other stable states diverged from the typical gene expression patterns of activated monocytes and macrophages.

Regarding the regulatory interactions between TFs and their target genes displayed in figure 1, we observed that several TFs exert only a few interactions. For example, CREB solely activates ALOX15, and no other target gene. Furthermore, this regulatory graph contains very few specific moDC markers. Consequently, to complete this preliminary network, we decided to take advantage of the public epigenome and transcriptome datasets to infer novel regulatory interactions and integrate them into our logical model (a proof of concept of this approach can be found in [17]).

### 2.2. Epigenomic data analysis helps to unravel relevant transcriptional regulatory interactions

In order to complete our model of the regulatory network controlling the differentiation of monocytes into moDCs, we included the TFs known to be activated by CSF2 and IL4 signals in moDCs, together with established monocyte markers. Moreover, we included information regarding the differentiation of monocytes into macrophages, which occurs when monocytes are treated with CSF2 alone [21]. In short, we (i) used monocyte, moDC and macrophage epigenome data to define chromatin states, (ii) defined genomic regions likely to be involved in the regulation of the genes included in the model, and (iii) searched for putative TFs binding sites in these regions.

**Figure 2.**
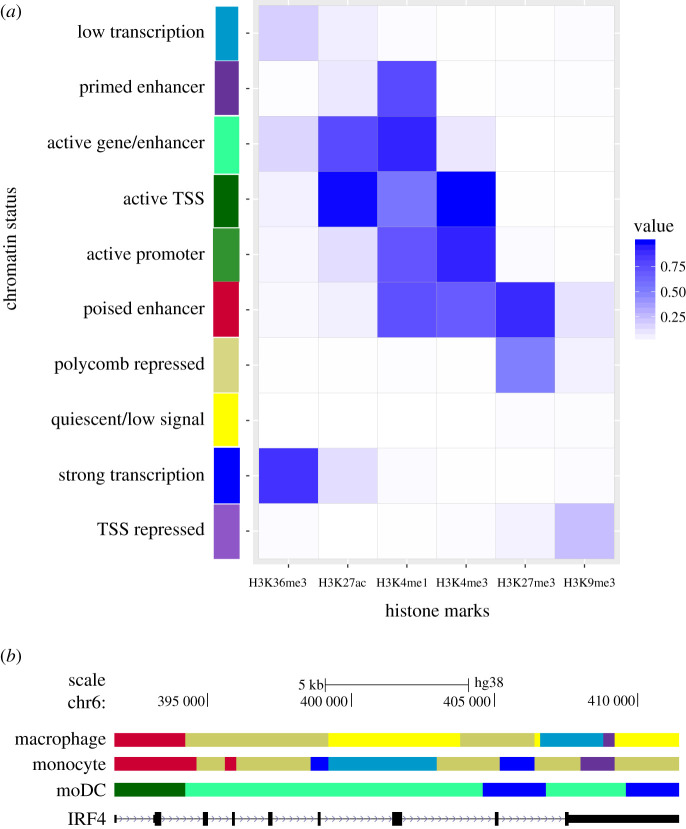
Epigenomic annotations of monocytes, moDCs and macrophages. (*a*) Heatmap showing the histone mark enrichment in each of the states determined with ChromHMM. (*b*) The genomic region coding for IRF4 is displayed in the UCSC genome browser. For each cell type, the ChromHMM analysis generates a specific segmentation, where each colour represents a chromatin state defined in the heatmap shown above. The annotation of chromatin state was derived from biological knowledge. Note that for this example, active gene marks (in green) are present only in the moDC track.

We analysed ChIP-seq data from the Blueprint consortium for six histone marks (H3K4me1, H3K4me3, H3K27ac, H3K36me3, H3K9me3 and H3K27me3) in monocytes, moDCs and macrophages derived from monocytes. We then used ChromHMM [22] to annotate the epigenome in each cell type based on these data. The resulting genomic segment states were classified into 10 categories: quiescent/low signal, polycomb repressed, poised regulation, active TSS, active promoter, Primed enhancer, active gene/enhancer, low transcription, TSS repressed and strong transcription (figure 2*a*). As expected, it is possible to visualize clear differences in the epigenome of moDCs versus monocytes when exploring genes with specific cell expression in a genome browser. For example, the gene coding for IRF4, a TF that mediates the differentiation of moDCs, is active in moDCs, while it is poised in macrophages and monocytes (figure 2*b*).

In the following step, we selected the segments corresponding to promoter and enhancer states: active TSS, repressed TSS, active gene/enhancer and poised regulation. These regulatory regions were used to predict binding sites for the TFs known to be activated by the CSF2 and IL4 pathways (figure 1), using position-weight matrices collected from the Jaspar database [19], with the pattern-matching tool *matrix-scan* [20] from the RSAT suite [23]. This led us to predict novel transcription factor binding sites presumably involved in the regulation of specific gene markers for moDCs, monocytes and macrophages (table 1), thereby enabling us to complete the regulatory network controlling monocyte to moDC differentiation.

**Table 1.** Cell type-specific gene markers selected to be added to the model. Based on the epigenome analysis, we identified novel regulatory interactions pointing to candidate genes for inclusion in our model.

cell type	gene	reference
moDCs	TLR8	[24]
moDCs	TLR7	[24]
moDCs	TLR6	[25]
moDCs	TLR4	[24]
moDCs	TLR3	[24]
moDCs	NCOR2	[9]
moDCs	DEC205 (LY75)	[24]
moDCs	DCIR (CLEC4A)	[24]
moDCs	CD83	[26]
moDCs	CD48	[9]
moDCs	CD226	[9]
moDCs	CD209	[24]
moDCs	CD1C	[9]
moDCs	CD1B	[27]
moDCs	CD1A	[28]
moDCs	CD141 (THBD)	[27]
moDCs	ITGAX (CD11C)	[9,29]
moDCs	CCL22	[9]
moDCs	CCL2	[30]
monocyte	CD14	[31]
monocyte	SELL	[9]
macrophage	CD163	[9]
macrophage	CCDC151	[32]
macrophage	MERTK	[9]
macrophage	CD206	[9]

**Figure 3.**
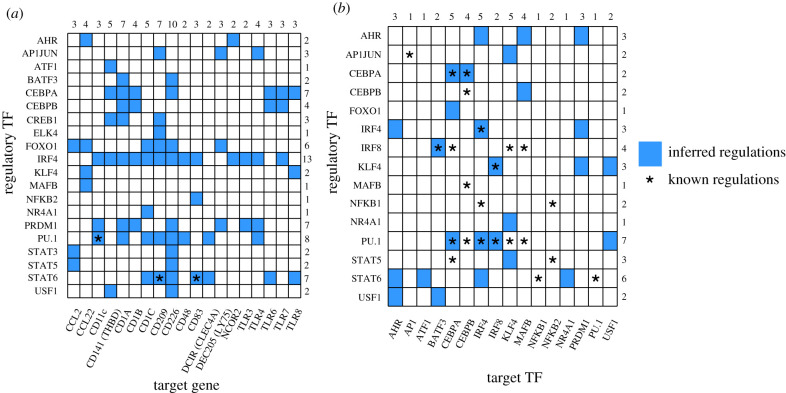
Predicted transcriptional regulatory interactions by TFs activated through CSF2 and IL4 signalling cascades. Position-weight matrices (from Jaspar database) for relevant TFs were used to predict binding sites in selected regulatory regions (based on chromatin state annotations) of specific genes. (*a*) The rows correspond to the regulatory TFs and the columns to the moDC-specific target genes. (*b*) The rows list the regulatory TFs and the columns list the target TFs. Turquoise coloured squares denoted the prediction of binding sites for the specified TF in the corresponding target gene region. Asterisks emphasize previously reported regulatory interactions (for the corresponding references, see electronic supplementary material, table S2). For each TF, the number of targets is reported on the right. The number of TF regulating each target gene is reported on the top of the corresponding column.

For 13 out of 19 genes related to moDC phenotype, we detected putative binding sites for IRF4 (figure 3*a*) into putative regulatory regions (cf. ChromHMM analysis) located near to the TSS of the corresponding genes. Interestingly, this led us to corroborate the central role of IRF4 in moDC differentiation. Indeed, we predicted that IRF4 possibly regulates several TLR genes (TLR3, 4 and 7), which play a crucial role in antigen recognition in myeloid cells and are thus relevant for moDC. Furthermore, we predicted that TLR6 and TLR8 are regulated by STAT6, another essential TF in moDCs [7], we acknowledge that TLRs genes are not specific for moDCs; however, their transcriptional regulatory mechanisms are not fully understood and our results provided useful insights for future studies. In addition, we predicted that the genes encoding for the trans-membrane proteins CD1A, CD1B and CD1C are regulated by IRF4, as well as by other TFs (PU1, PRDM1, NR4A1 and CEBPA) related to moDC differentiation. Furthermore, we predicted that the gene coding for CD48, a costimulatory molecule involved in T cell activation, is regulated by PU1, which is known to participate in the differentiation of STEM cell progenitors into leucocytes [33]. We also analysed the non-redundant peaks available from Remap and identified several concordances with our results (electronic supplementary material, table S6). We also looked for regulatory interactions between the identified TFs. In particular, we predicted regulatory interactions from PU1 onto CEBPA, IRF4 and IRF8. We also predicted that AHR regulates IRF4, MAFB and PRDM1, which represent interesting candidates to assess experimentally. Figure 3*b* summarizes the regulatory interactions between TFs that compose our final logical model.

### 2.3. Integration of predicted novel regulatory interactions improves model accuracy

**Figure 4.**
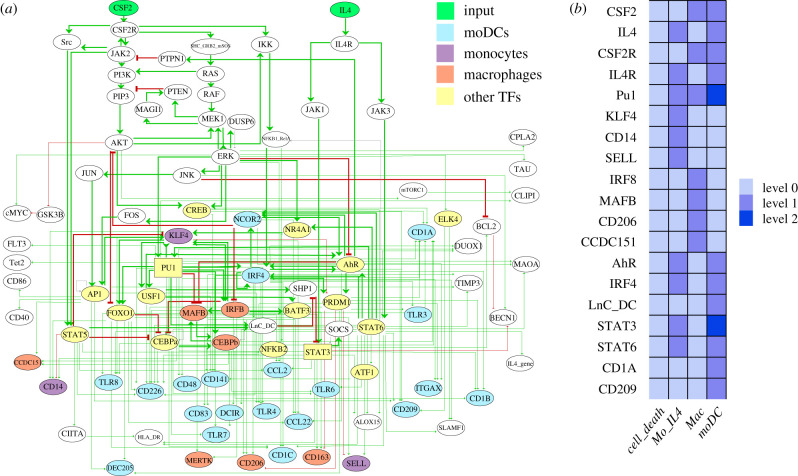
Logical model for the *in vitro* differentiation of monocytes into moDCs. (*a*) Ellipsoid nodes are associated with Boolean components (taking the values 0 and 1), whereas the two rectangular nodes are associated with ternary components (taking the values 0, 1 and 2). The green nodes at the top represent the inputs (CSF2 and IL4), the yellow nodes denote TFs, blue nodes correspond to moDC-specific genes, orange nodes to macrophage-specific genes and purple nodes to monocyte specific genes. Green and red arcs denote positive and negative interactions, respectively. (*b*) Stable states of the model and matching cell types (only the most relevant nodes are shown). The first column corresponds to the final outcome in the absence of both IL4 and CSF2, i.e. cell death of the monocytes. The second column corresponds to the stimulation of monocytes by IL4. The third column corresponds to the macrophage outcome, in the presence of the sole CSF2. Finally, the fourth column corresponds to moDC commitment, in the presence of both IL4 and CSF2, where STAT3 reaches level 2 in the presence of the long non-coding RNA LnC-DC, and PU1 reaches also level 2, which is required to turn-off MAFB during moDC commitment (for a complete listing of node values at the four stable states, see electronic supplementary material figure S1).

We integrated the selected gene markers for each cell type with the predicted regulatory TFs into our model and connected them with the regulatory interactions reported in figure 3. Using this extended regulatory graph (figure 4*a*) together with relevant Boolean rules, we computed the stable states, which consistently recapitulated the main cell fates (see electronic supplementary material, table S3).

Our revised model is characterized by four stable states (figure 4*b*; electronic supplementary material, figure S1). The first stable state, in the absence of IL4 and CSF2 activation, corresponds to cell death, which is the expected outcome in this situation. The second stable state is obtained upon IL4 activation and corresponds to the monocyte signature, with KLF4, SELL and CD14, in the ON setting. The third stable state is obtained for CSF2 activation and corresponds to a macrophage signature, with MAFB, IRF8, CCDC151 and CD206 ON. Finally, the last stable state is obtained when both CSF2 and IL4 are activated and corresponds to the moDC signature*,* with IRF4, STAT6, CD1A and CD209 all ON. Noteworthy, this analysis recapitulates the fact that PI3K signalling is inhibited during moDC commitment, as previously reported by van de Laar *et al*. [6].

**Figure 5.**
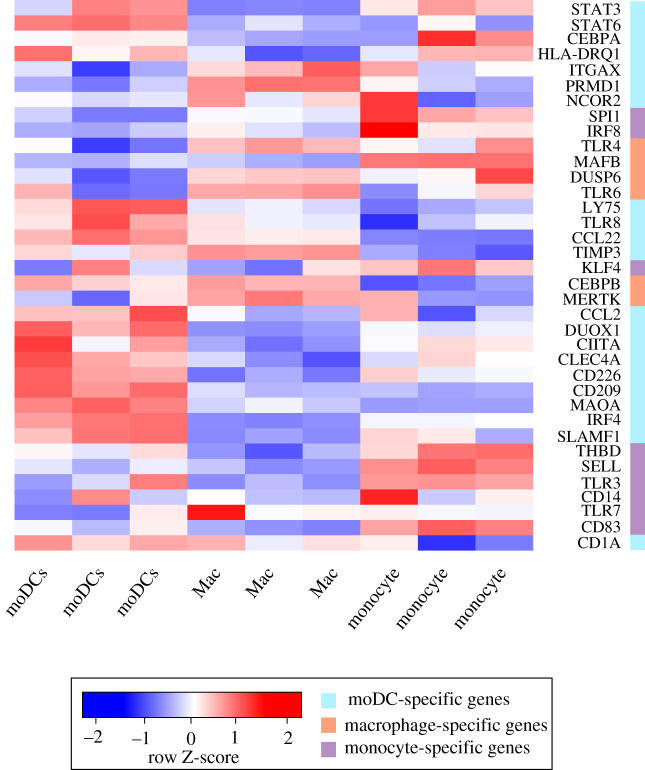
Heatmap showing differentially expressed genes between cell types after hierarchical clustering based on differential expression. The first three columns correspond to moDCs, the following three columns to macrophages and the last three columns to monocytes (each triplet of columns represent biological replicates). The colour-coded *Z*-score indicates the level of differential expression for each gene. Cell types are annotated based on known gene expression on the right, with light blue denoting moDCs expression, orange denoting macrophages and purple denoting monocytes.

To validate the different expression signatures, we analysed RNA-seq data from monocytes, moDCs and macrophages. Figure 5 displays the differential expression of the genes included in the model. Interestingly, we found two main clusters of genes highly expressed in moDCs, but downregulated in macrophages. These moDC differentially expressed genes include those coding for the TFs STAT3, STAT6, CEBPA and IRF4, which participate in moDC differentiation, as well as those for CD209, MAOA and SLAMF1, which are specific markers for moDCs. Additionally, monocytes display high expressions of the genes coding for KLF4, IRF8, SELL and CD14.

In the next step, we took advantage of the CoLoMoTo toolbox to recapitulate documented cellular commitment experiments.

### 2.4. Model simulations recapitulate the main aspects of cellular commitment to differentiation

We imported our model into the CoLoMoTo environment to ease further analyses with complementary software tools such as Pint, BioLQM and MaBoSS [34]. The integration of all analyses can be found in a Jupiter (python) notebook (available at http://ginsim.org/model/monocytes-to-dc) further ensures reproducibility.

We used the tool BioLQM to compute the trap-spaces for the wild-type situations [35]. As trap-spaces provide approximations of cyclic attractors, we could thereby verify that the model does not generate any cyclic attractors.

**Figure 6.**
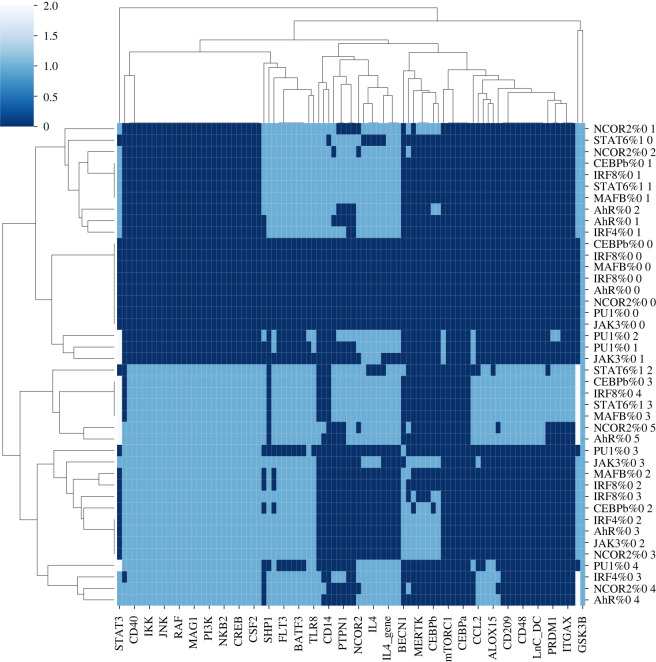
Clustered heatmap of the stable states obtained for all the perturbations considered. Each row represents one stable state for one perturbation. For example, ‘NCOR2%0 1’ denotes a knock-out of NCOR2 and corresponds to the first stable state obtained for this condition. Each column represents one of the 32 selected nodes of the logical model.

 We continued to use BioLQM [35] to assess the behaviour of the model for nine single gene losses-of-function (affecting IRF4, STAT6, PU1, IRF8, MAFB, NCOR2, AHR, JAK3 and CEBPB, respectively) that have been reported in the literature to affect the differentiation process. Table 2 summarizes the results obtained for these perturbations, while figure 6 shows the activity of each node for each perturbation at the stable states. These results qualitatively replicate the behaviour of each of the documented mutants. We further simulated the knock-out of each TF from figure 3*a* and identified that BATF3, FOXO1, PU1 and USF1 affected the three final cell types (electronic supplementary material, table S7).

**Table 2.** Perturbations tested in the model of monocyte to moDC differentiation.

protein	function	phenotype extracted from references	perturbation simulated	model phenotype
IRF4	transcription factor	monocytes were infected using lentiviral vectors containing shRNA against IRF4, silenced IRF4 induced a dramatic reduction of moDCs [11]	loss of function	lack of most of moDC-specific markers
STAT6	transcription factor	the ectopic expression of STAT6 in monocytes, resulted in increased levels of the DC-specific marker DC-sign, following CSF2 stimulation, and without IL4 [7]	gain of function	STAT6 is almost sufficient to archive moDCs differentiation
PU1	transcription factor	inducible constructions of PU1, and MAFB were used to infect monocytes. In cells with PU1 induced DCs, MafB differentiated macrophages [36]	loss of function	abolish moDCs, and macrophage phenotype commitment
IRF8	transcription factor	introduction of KLF4 into an Irf8-/- myeloid progenitor cell line induced a subset of IRF8 target genes and caused partial monocyte differentiation [30]	loss of function	abolish KLF4 expression, and the entire macrophage differentiation
MAFB	transcription factor	silencing of MAFB resulted in a strong decrease in mo-Macs, and an increase in mo-DC differentiation [36]	loss of function	moDCs differentiation is normal, Mac differentiation is abolished
NCOR2	transcriptional regulator	NCOR2 silencing resulted in 1834 variable genes that correspond with IL4 signature genes [9]	loss of function	lack of moDC-specific markers
AHR	transcription factor	AHR silencing reduced mo-DC differentiation while slightly increasing mo-Mac [11]	loss of function	lack of every moDC-specific markers, Mac differentiation is normal
JAK3	tyrosine-protein kinase	STAT6 phosphorylation disappeared following JAK3 inhibition. In the case of Macs, we did not observe STAT6 phosphorylation, given the lack of stimulation of JAK3 [7]	loss of function	Mac phenotype with CSF2, and IL4. Mac differentiation is not affected
CEBPB	transcription factor	in the absence of CEBPb in monocytes CEBPb-KO, only a very low amount (5%) of this Mac-like morphology was seen, and most of the cells stayed round [37]	loss of function	lack of some specific macrophage markers

**Figure 7.**
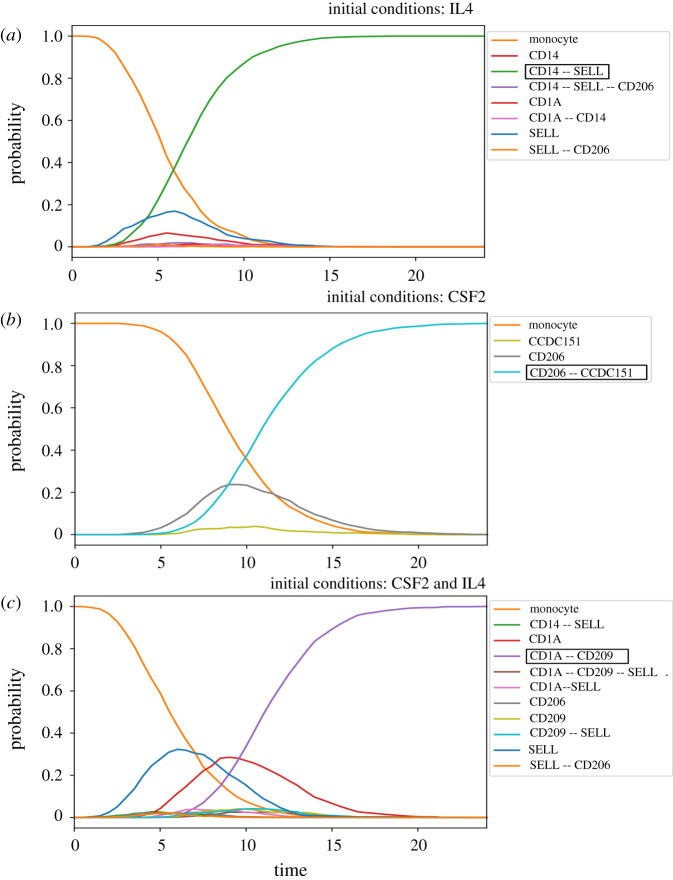
Probabilities of each combination of activated selected markers (among CD1A, CD14, CD206 CD209, SELL) (y-axis) over time (x-axis). Each curve represents mean values over 1000 stochastic simulations starting from a quiescent monocyte state (IRF8, FOS, AP1, CREB, ELK4 are ON, active): (*a*), (*b*) and (*c*) correspond to the scenarios where IL4, CSF2, and both CSF2 and IL4 are activated, each leading to one final activity pattern corresponding to stimulated monocytes, macrophages and moDCs, respectively.

We then used the tool Pint [38] to verify the reachability of the stable states corresponding to the correct cell commitment for each combination of CSF2 and ILF4, starting from a quiescent Monocyte state. Next, we used the stochastic Boolean simulation tool MaBoSS to generate mean temporal curves, starting from initial conditions corresponding to quiescent Monocytes, towards the stable state reached in the presence of the corresponding combination of active CSF2 and ILF4 [39]. In figure 7, we can observe several curves initially growing but later decaying. These curves most likely represent transient cellular states during the conversion of cells from their initial state to the attracting state reached at the end of the simulations.

## 3. Discussion

The construction of logical models traditionally relies on manual curation of the literature on a biological system of interest. In this work, we further took advantage of public ChIP-seq and RNA-seq data from the Blueprint consortium [40] to delineate in more detail the network driving the differentiation of monocytes into moDCs. We were able to fill in various gaps in the regulatory network, which allowed us to reach a better understanding of this differentiation process. Noteworthy, this led us to predict 102 novel interactions, which were validated *in silico* through our simulations, and are amenable to further experimental tests.

In particular, we delineated a series of target genes presumably important for the differentiation of monocytes into moDCs. Some TFs are already well known, such as IRF4, AHR, STAT6 and PU1 [7,11,29]. Our analysis recapitulates several key features regarding the expression of the corresponding genes, such as a high expression of IRF4 and STAT6 genes in moDCs. We further validated the results obtained by Vento-Tormo *et al.* [7] indicating that STAT6 is required for moDCs differentiation; according to our model analysis, STAT6 is indeed required for moDC differentiation, but not for macrophage commitment (figure 6).

We were also able to predict novel TF targets that are relevant for this process, regulated by FOXO1, CEBPA, AP1 and PRDM1. We predict that FOXO1 regulates at least six moDC genes, while CEBPA could regulate at least seven of them. Furthermore, AP1 presumably regulates TLR4, DEC205 (LY75) and CD209 (DC-SING), which are associated with antigen-presenting cells. CREB1 is also presumably involved in the regulation of moDC genes, through the activation of CD141 and CD1A. We also predict for the first time that NR4A1 could regulate CD1C, a protein found at the surface of moDCs.

We further reviewed data recently published on the predicted TF-gene interaction considered in our model, and we found that some of these interactions have been recently experimentally confirmed. In particular, the regulation for the ITGAX gene was shown to be regulated by PU1 and IRF4 [29], as predicted by our epigenomic data analysis.

In summary, this study represents the first effort to integrate the current knowledge on monocytes to moDCs differentiation *in vitro* and should foster our understanding of this process. Additionally, we unravelled novel transcriptional regulatory links presumably involved in this differentiation process.

## 4. Material and methods

### 4.1. Model implementation and simulations

Using the software GINsim [16], we integrated previously described signalling pathways activated when monocytes are cultured with CSF2 and IL4 (all the corresponding studies are referenced in the GINsim model as node annotations, and further listed in electronic supplementary material, table S3). We also reviewed the literature on the differentiation of monocytes into moDCs. The logical model was built using GINsim v. 3.0 [41], where nodes represent genes or proteins, and edges represent regulatory interactions between them, which can be negative or positive (or sometimes dual). In general, each node can take two values, zero or one, but in special cases, one may need to consider different qualitative levels of activation (e.g. STAT3 expression is activated by JAK1, but the presence of LnC-DC leads to a further increase of STAT3 expression). For such special cases, it is possible to use multilevel nodes, e.g. ternary variables enabling an additional level of activation (hence taking the values 0, 1 and 2). Logical rules are associated with each component of the network, combining literals (i.e. regulatory variables with specific values) with the classical Boolean operators AND (&), OR (|) and NOT (!), thereby defining in which conditions each of these components can be activated or shut down. When considering novel predicted regulatory interactions, we started by setting a generic rule such which established that any activator component is sufficient to activate its target (OR), provided that all inhibitors are absent (AND, NOT). These rules were then refined to match the reported cell gene expression patterns.

### 4.2. ChIP-seq data analysis

Raw fastq files from ChIP-seq experiments were retrieved from the Blueprint Consortium [31] data access portal (http://dcc.blueprint-epigenome.eu/#/datasets) with dataset identifiers EGAD00001001552, EGAD00001002484, EGAD00001002485, EGAD00001001576 and EGAD00001002504. We processed datasets for six histone marks (H3K4me1, H3K4me3, H3K27ac, H3K36me3, H3K9me3 and H3K27me3), in triplicates, from human monocytes, macrophages and moDCs. We performed quality control of read sequences with FastQC/0.11.3 tool [42], used Trimmomatic/0.33 [43] to improve their quality, and then mapped them with bowtie 2-2.2.6 [44] to the human hg38 reference genome with default parameters (--sensitive -- phred33). A second quality control was performed after alignment, using ENCODE QC (electronic supplementary material, table S4), which consists of three major tests: NRF (non-redundant fraction) > 0.5, PBC1 (PCR Bottleneck coefficient 1) > 0.5 and PBC2 (PCR Bottleneck coefficient 2) > 0.5 [45]. IDR analysis [45] was performed for replicate control with all replicates successfully passing this test.

### 4.3. Chromatin states definition

We used ChIP-seq data for six histone marks (H3K4me1, H3K4me3, H3K27ac, H3K36me3, H3K9me3 and H3K27me3) for each cell type (monocytes, macrophages and moDCs), with their respective input control. Chromatin states were defined using ChromHMM [18] v. 1.12 [46] with the recommended parameters (BinarizeBed -b 200, assembly hg38), and specifying 10 hidden states for the hidden Markov model. Each chromatin state was annotated based on the probability of appearance of the different marks (e.g. H3K27ac-Enhancers, H3Kme1-Enhancers, H3K4me3-Promoters, H3K27me3-Repressive, H3K9me3-Repressive and H3K36me3-Transcribed [47]). We then assessed the enrichment in different kinds of genome loci (CpGIsland, RefSeqExon, RefSeqGene, RefSeqTES, RefSeqTSS and RefSeq2kb) to the specific chromatin states. Integrating this information, we were able to assign a functional description to each state. Next, we focused on active TSS, repressed TSS, active gene/enhancer and poised regulation regions, based on RefSeqTSS annotations.

### 4.4. Search for transcription factor binding site using matrix-scan

Based on the literature, we identified 22 TFs participating in the differentiation of monocytes to macrophages or moDCs. We retrieved one Position-Specific Scoring Matrix (PSSM) for each of these 22 TFs (electronic supplementary material, table S5) from the JASPAR 2018 database human collection [19]. We performed pattern-matching searches for TF motif instances using the 22 PSSMs in the selected chromatin regulatory regions, active TSS, repressed TSS, active gene/enhancer and Poised regulation in the vicinity of the TSS annotated in RefSeqTSS (see ChromHMM analysis above). For this task, we used the tool *matrix-scan* [20] from the RSAT suite [23] with the following main parameters: background model of Markov order 1, and stringent thresholds of *p* ≤ 10^−5^, and score 1 (-markov 1 -lth score 1 -uth pval 1 × 10^−5^). In order to assess our results regarding the number of predicted interactions discovered in figure 3*a*, we generated 100 groups of 20 randomly selected genes, selected their regulatory regions using ChromHMM, used matrix-scan (as described above) with the same collection of motifs and performed a hypergeometric test in the electronic supplementary material, figure S2.

### 4.5. RNA-seq analyses

Raw fastq files from RNA-seq experiments were retrieved from the Blueprint Consortium [40] data access portal (http://dcc.blueprint-epigenome.eu/#/datasets) with dataset identifiers: EGAD00001002308, EGAD00001001506, EGAD00001002526, EGAD00001002507 and EGAD00001001582. Blueprint monocytes samples were obtained from the same laboratory from healthy volunteers using positive selection, then the same samples were treated with CSF2 and IL4 for 6 days (monocyte purification: https://www.blueprint-epigenome.eu/UserFiles/File/Protocols/UCAM_BluePrint_Monocyte.pdf).

For this analysis, we used the methods described in Law *et al.* [48]. In brief, we performed quality control with FastQC/0.11.3 [49], pseudo-alignment and count determination with Kallisto 0.43.1 [50] using the release-90 from Ensembl (ftp://ftp.ensembl.org/pub/release-90/fasta/homo_sapiens/cdna/Homo_sapiens.GRCh38.cdna.all.fa.gz) to create our index with the following command: kallisto index -i index_kallisto_hsap_90_cdna --make-unique Homo_sapiens.GRCh38.cdna.all.fa.gz. Counts were assigned to genes using Tximport 1.14.0 [51] and were processed from raw-scale to counts per million (CPM), and then they were transformed to log-CPM. Genes with expression values below 1 were removed. Then we normalized raw library sizes using the *calcNormFactors* function from edgeR library in R. Afterwards, we performed a differential gene expression analysis with edgeR 3.28.0 [52]. Finally, we used heatmap.2 from the gplots library to plot the genes found in our model (figure 5).

### 4.6. Colomoto analysis

In order to foster reproducibility, we used the CoLoMoTo toolbox [18] that integrates several logical modelling tools, including GINsim, bioLQM, Pint and MaBoSS. We used GINsim to compute the stable states and bioLQM to identify trap-spaces approximating cyclic attractors. The computation of mean stochastic temporal trajectories was performed using MaBoSS [39]. The GINsim model and the CoLoMoTo notebook are available on the GINsim repository (http://ginsim.org/model/monocytes-to-dc), as well as a dedicated git repository (https://github.com/karenunez/moDC_model_differentiation). The results can be replicated with the CoLoMoTo Docker image following the instructions provided at http://colomoto.org/notebook.

### 4.7. Generation of the figures

Figure 1 and 4*a* were generated with the GINsim software. The plots in figures 2*a*, 3*a*,*b* and 4*b* were generated with the ggplot2 library from R. Figures 6 and 7 were extracted from the CoLoMoTo notebook developed for this study.

## Data accessibility

Supplementary files, including the logical model of monocyte to DCs differentiation, are available at https://github.com/karenunez/moDC_model_differentiation.
